# Assessment of the transmission of live-attenuated chikungunya virus vaccine VLA1553 by *Aedes albopictus* mosquitoes

**DOI:** 10.1186/s13071-025-06789-w

**Published:** 2025-05-12

**Authors:** Andrea Fritzer, Andreas Suhrbier, Leon E. Hugo, Bing Tang, Greg Devine, Sandra Jost, Andreas L. Meinke

**Affiliations:** 1 Valneva Austria GmbH, Campus Vienna Biocenter 3, 1030 Vienna, Austria; 2 Inflammation Biology, QIMR Berghofer Medical Research Institute, Brisbane, QLD 4029 Australia; 3 Mosquito Control, QIMR Berghofer Medical Research Institute, Brisbane, QLD 4029 Australia

**Keywords:** Alphavirus, Chikungunya, IXCHIQ, VLA1553, Mosquito, Transmission, Vaccine, Mouse

## Abstract

**Background:**

Chikungunya virus (CHIKV) is a mosquito-transmitted, arthritogenic alphavirus that causes sporadic outbreaks of often debilitating rheumatic disease. The recently approved CHIKV vaccine, IXCHIQ, is based on a live-attenuated CHIKV strain (VLA1553), with viraemic vaccine recipients theoretically able to transmit VLA1553 to mosquitoes with ensuing onward transmission. We thus evaluated VLA1553 transmission from artificial blood meals to *Aedes albopictus* mosquitoes, and onward transmission to mice.

**Methods:**

Female *A. albopictus* mosquitoes were fed on defibrinated sheep blood containing wild-type CHIKV (viral titre: 7.50 log_10_CCID_50_/mL) or VLA1553 (viral titres: 7.85, 5.72, 4.58, and 3.79 log_10_CCID_50_/mL). Viral titres in mosquito bodies and saliva were determined using CCID_50_ assays 7–8 days after the blood meal. After providing CHIKV or VLA1553 (viral titres ~ 7–8 log_10_CCID_50_/mL) in blood meals to mosquitoes, infected mosquitoes were fed on highly susceptible *Irf3/7*^−/−^ mice (*n* = 3 per group). Data were re-analysed using the same reverse transcription quantitative polymerase chain reaction (RT-qPCR) as for an earlier VLA1553 phase 1 clinical trial, to allow correlations between blood meal titres and viraemia in vaccine recipients.

**Results:**

Mosquito body viral titres were significantly higher (*P* < 0.0001) for CHIKV versus VLA1553-fed mosquitoes at blood meal viral titres of ~ 7–8 log_10_CCID_50_/mL. Mosquito body VLA1553 titres decreased with reducing blood meal titres, but there was no dose-dependent effect on saliva viral titres. No dissemination to salivary glands was seen at blood meal titres ≤ 3.875 log_10_CCID_50_/mL. CHIKV-fed mosquitoes were able to transmit virus, and induce viraemia in, 3/3 *Irf3/7*^−/−^ mice via mosquito bites. In contrast, 0/3 *Irf3/7*^−/−^ mice became infected after bites from VLA1553-fed mosquitoes. RT-qPCR comparisons with phase 1 clinical data for VLA1553-vaccinated individuals indicated that VLA1553 viraemia was at or below the aforementioned threshold for transmission.

**Conclusions:**

The evidence presented herein argue that the low viraemia in VLA1553-vaccinated individuals would mitigate against transmission. In addition, replication of VLA1553 in mosquito bodies was also significantly attenuated. Overall, mosquito-borne transmission of VLA1553 from vaccinated individuals to others appears improbable.

**Graphical Abstract:**

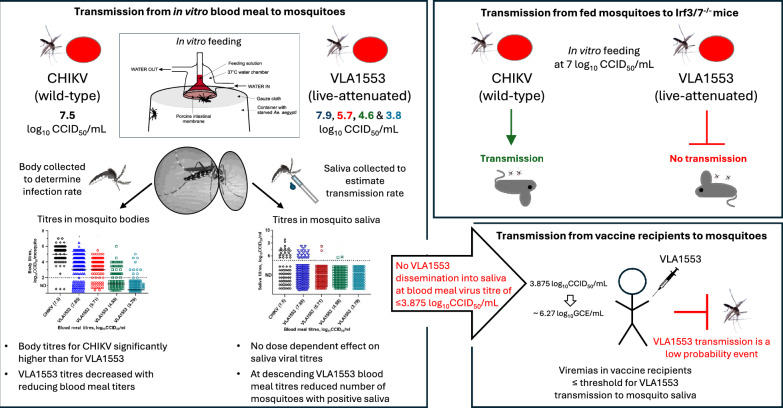

## Background

Increasing international travel and intensification of trade, the spread of mosquito vectors and global warming are all contributing to increasing risks of arthropod-borne virus (arbovirus) outbreaks [1–3]. Pathogenic flaviviruses and alphaviruses present the major arboviral burden to human and animal health globally [4].

Chikungunya virus (CHIKV) is a mosquito-transmitted alphavirus (enveloped with single-stranded RNA genome) that is transmitted mainly by *Aedes aegypti* and *Aedes albopictus* mosquitoes [2, 5, 6]. Since its first isolation in 1952 in Tanzania, CHIKV has caused sporadic outbreaks of disease every 2–50 years [7]; however, in 2004, the largest recorded epidemic of CHIKV began and expanded across four continents, affecting > 100 countries and causing > 10 million cases [2]. Outbreaks continue, with ~ 450,000 CHIKV cases and over 160 deaths reported worldwide in 2024 [8]. CHIKV is also appearing in new locations where it has not previously been seen [9].

The disease caused by CHIKV is characterised by a constellation of acute and chronic, primarily rheumatic, manifestations, but can also present with a spectrum of atypical and severe manifestations, with a mortality rate of 0.024–0.7% [2]. CHIKV disease is also associated with an increased risk of all-cause mortality [10]. Reported rates of chronic disease vary widely, with, for instance, 0.3–21% of patients still suffering, primarily from arthralgia, at 1 year post-infection [11, 12]. However, poor attention to differential diagnoses often complicates such studies [2] and can mean that other treatable rheumatic conditions are overlooked [13]. The burden of disease has been estimated to be > 106,000 disability-adjusted life years (DALYs) between 2010 and 2019 [14], and the economic impact was estimated to be US$9.3 billion during the period 2003–2020 [15]. A recent study calculated a total of 18.7 million CHIKV cases in 110 countries between 2011 and 2020, causing 1.95 million DALYs, with an economic burden of US$2.8 billion in direct costs and US$47.1 billion in indirect costs worldwide [16].

In the absence of licensed anti-viral treatments for CHIKV infection or particularly effective treatments for CHIKV disease [4, 17], Valneva developed a live-attenuated vaccine (VLA1553) for active immunisation against CHIKV infection [18]. Following successful preclinical [19, 20] and clinical evaluations [21–26], VLA1553 was approved by the Food and Drug Administration (FDA) in November 2023 [27], by Health Canada in June 2024 [28] and by the European Commission in July 2024 [29].

Like other live-attenuated arboviral vaccines [30, 31], VLA1553 has the potential to be transmitted from viraemic vaccine recipients to mosquitoes, with infected mosquitoes then potentially able to transmit VLA1553 to others via a subsequent mosquito bite. For this to occur, the vaccinated individual would need to develop a sufficiently high viraemia for VLA1553 in the blood meal to infect the mosquito midgut [32, 33]. The midgut escape barrier [34] would then need to be overcome for the virus to enter the mosquito body. Thereafter, the salivary gland infection and escape barriers would need to be overcome and sufficient titres delivered to allow onwards transmission to new human hosts [32, 35]. The titre of virus in the blood meal plays a key role in determining how efficiently an arbovirus can pass through these barriers and complete the transmission cycle. Transmission is largely lost when blood meal titres fall below a certain threshold, with titre versus transmission efficiency relationships available for CHIKV [33, 36, 37] and dengue virus [38, 39].

Herein, we evaluate the transmission of a Réunion Island CHIKV patient isolate (LR2006 OPY1) [40] (hereafter referred to as CHIKV) and VLA1553, from artificial blood meals to *A. albopictus* mosquitoes. *A. albopictus* has a high susceptibility to infection by Indian Ocean lineage viruses that have the E1:A226V mutation [41, 42], such as LR2006 OPY1, the parental strain of VLA1553 [24].

We also evaluate the ability of CHIKV- and VLA1553-fed *A. albopictus* mosquitoes to infect *Irf3/7*^−/−^ mice via mosquito bites. This mouse strain does not express interferon response factors 3 and 7 and is therefore highly susceptible to CHIKV infection owing to defective type I interferon responses [43, 44].

## Methods

All mouse work was undertaken in accordance with the Australian Code for the Care and Use of Animals for Scientific Purposes as defined by the National Health and Medical Research Council of Australia and was approved by the Queensland Institute of Medical Research (QIMR) Berghofer Medical Research Institute (MRI) Animal Ethics Committee. Details of agistment conditions have been described previously [45].

All work with infectious virus was conducted in a PC3 (biosafety level 3) facility at the QIMR Berghofer MRI (Department of Agriculture, Fisheries and Forestry, certification Q2326 and Office of the Gene Technology Regulator certification 3,445). Breeding and use of genetically modified (GM) mice was approved under a Notifiable Low Risk Dealing (NLRD) identifier: NLRD_Suhrbier_Oct2020: NLRD 1.1(a). The mice were held under standard animal house conditions (details provided previously [45]).

VLA1553 was imported via an Australian Government Department of Agriculture import permit no. 0001554905.

### *A. albopictus* mosquitoes

A colony of *A. albopictus* mosquitoes was established from eggs collected on Hammond Island (Torres Strait, Australia) in May 2014, with additional wild-caught mosquitoes included in 2015. The colony was maintained in a climate-controlled insectary (27 °C, 70% relative humidity, and 12 h light:12 h dark cycling with 30-min crepuscular periods) at the QIMR Berghofer Medical Research Institute, Brisbane, Australia. Eggs were hatched by flooding in rainwater. Larvae were reared in rainwater in plastic trays at a density of ~ 300 larvae per tray. Larvae were fed ground TetraMin Tropical Flakes fish food (Tetra, Melle, Germany) ad libitum. Pupae were collected and placed in a container of rainwater inside a 30 × 30 × 30 cm^3^ cage (BugDorm, MegaView Science Education Services Co., Taichung, Taiwan) containing cotton wool pledgets with a 10% sucrose solution.

Prior to feeding, mosquitoes (5–6 days old) were deprived of sucrose solution for 36 h. Female mosquitoes were sampled from the cage by placing a bottle of hot water beside the cage wall and aspirating females that probed against the bottle. Female mosquitoes were transferred to 750-mL plastic containers with gauze lids.

### CHIKV and VLA1553 blood meals for *A. albopictus* mosquitoes

Female *A. albopictus* mosquitoes were fed on defibrinated sheep blood (Life Technologies, Mulgrave, VIC, Australia) containing either CHIKV (LR2006-OPY1, GenBank KT449801) [46]) or VLA1553. Feeding was performed essentially as described previously [33], except that porcine intestinal membrane was used instead of bovine caecum lining. For the comparison between CHIKV and VLA1553 for transmission to *A. albopictus* mosquitoes, blood meals contained a high viral titre of ~ 7–8 log_10_CCID_50_/mL of CHIKV or VLA1553 (series 1 experiments). For determination of a threshold titre for VLA1553 transmission, blood meals contained descending VLA1553 titres (series 2 experiments). The experiments in each series were performed in triplicate, and ~ 100 fed mosquitoes were analysed for CHIKV and VLA1553 body and saliva infection. CHIKV was generated in C6/36 cells as described previously [47]. Mosquitoes were offered blood meals for 1 h via the porcine intestinal membrane using an artificial feeding apparatus (kept at 37 °C) as described previously [33]. Blood meal CHIKV/ VLA1553 titres were determined by CCID_50_ assays on blood meal samples taken before and after mosquito feeding. The actual blood meal virus titres in the membrane feeders were determined as the mean of the titres measured before and after feeding to verify that the pre-defined viral titres in the sheep blood were correct, and to ensure that the titre of viable virus remained stable during the feeding process. Engorged mosquitoes were anaesthetised with CO_2_ and placed on a Petri dish on wet ice, collected and maintained in an environmental chamber with the same climate control as described for the *A. albopictus* colony (Taiwan HiPoint Corporation, Kaohsiung City, Taiwan). The volume of blood meal taken up by the mosquitoes was not determined. The cages contained trays (10 × 15 cm^2^) with water (about 20% full) with damp filter paper (around the inside tray walls) to allow the mosquitoes to lay eggs, which provides a stimulus for the mosquitoes to take a second blood meal (required for the *Irf3/7*^−/−^ feeding experiments).

### The live-attenuated CHIK vaccine, VLA1553

VLA1553 stock was produced using Vero cells (African green monkey kidney epithelial cell line; ATCC CCL-81). To provide high virus titres in blood meals used for feeding of mosquitoes, all experiments at QIMR Berghofer MRI were performed with the VLA1553 material pooled after sucrose gradient centrifugation (SGP). The SGP material is a high-titre intermediate in the manufacture of VLA1553. The SGP material was produced under Good Manufacturing Practice conditions as a liquid frozen formulation and had a virus titre of 2.5 × 10^8^ TCID_50_/mL (8.4 log/mL), allowing the generation of blood meals with virus titres of ~ 7–8 log_10_ CCID_50_/mL.

### Virus titre determinations

*Mosquito bodies*. Viral titres in individual mosquito bodies were determined using CCID_50_ assays as described previously [33] 7–8 days after the blood meal, to coincide with the time when infection levels plateau [48]. Individual mosquitoes (anaesthetised and collected as described above) were placed in 2-mL screw cap vials with two zirconium silica beads and 500 μL of medium (Roswell Park Memorial Institute [RPMI] 1640, 2% FBS/FCS, 0.25 μg/mL amphotericin B and 10 mM HEPES). Mosquitoes were homogenised by shaking tubes for 1.5 min in a chilled block using a MiniBeadbeater-96 sample homogeniser (Biospec Products, Bartlesville, OK, USA) followed by centrifugation (twice top speed on a bench microfuge, 5 min, 4 °C, with 180° tube rotation). Samples were frozen at −80 °C. The titres reported for each individual mosquito represent the titres (CCID_50_/mL) in the 500-μL samples. Since an average mosquito has a weight of about 5 mg and assuming 80% water content, the estimated actual viral titres in the mosquito body fluid would be approximately 125-fold higher.

*Mosquito saliva*. Viral titres in saliva were determined by CCID_50_ assay. Saliva was collected essentially according to the method of Poole-Smith et al. [49], with slight modifications. Mosquitoes were anaesthetised with 15-s exposure to CO_2_, placed on wet ice, and the legs and wings were removed. The body was placed onto double-sided tape on a flat plate (at room temperature). The proboscis of each mosquito was then inserted into an individual microcapillary tube containing 10 µL of 10% aqueous sucrose (wt/vol) solution supplemented with 10% FBS for 20 min. Capillary tube contents were expelled into a 2-mL screw cap tube (Sarstedt), 45 µL of medium supplemented with 2% FCS was added, and tubes were frozen at −80° C.

The mean amount of salivary gland expectorant obtained from other studies for 11-day-old *A. aegypti* mosquitoes (similar to the age used in our study) has previously been shown to be 4.5 nL [50]. We assumed similar volumes were delivered by *A. albopictus* mosquitoes into the 10 µL of sucrose solution in our experiments. The titres determined to be present in the 10 µL of sucrose solution were thus adjusted to provide the estimated titres in the saliva by multiplying the titres determined to be in the 10 µL of sucrose solution by 2200 (10,000/4.5).

***CCID***_***50***_*** assay***. The CCID_50_ assays were undertaken as described previously [33, 47], and were performed using tenfold (body, blood meal) or fivefold (saliva) serial dilutions of the virus-containing solution in 96-well plates in duplicate on C6/36 cells (about 2 × 10^4^ cells/well). After 3 days of incubation at 28 °C, 25 µL from each well was transferred into an equivalent parallel well of another 96-well plate containing Vero cells (about 2 × 10^4^ cells/well). This two-stage process (C6/36 titration/amplification followed by detection in Vero cells) increases the sensitivity and decreases the variability of the CCID_50_ assay for different viral strains [47]. After incubation of the Vero cell containing plates for 4 days at 37 °C, the plates were stained with Crystal Violet to visualise cytopathic effects in the Vero cells. The virus titre was calculated using the Spearman and Kärber method [51]. The limits of detection (LOD) were ~ 2 log_10_CCID_50_/mL for mouse sera and mosquito body homogenates, and ~ 5.3 log_10_CCID_50_/mL for saliva.

### Transmission of CHIKV versus VLA1553 to ***Irf3/7***^***−/−***^ mice by infected mosquitoes

Feeding of CHIKV- or VLA1553-fed *A. albopictus* mosquitoes on naïve *Irf3/7*^−/−^ mice [43] (three per group) was undertaken as described previously [33]. Briefly, mosquitoes were membrane fed on artificial blood meals (using defibrinated sheep blood) and left for 8 days (and allowed to lay eggs) and were then fed on the shaved belly area of isoflurane anaesthetised *Irf3/7*^−/−^ mice. Feeding of the mosquitoes on the mice was undertaken for 40 min to 1 h. The numbers of engorged mosquitoes per mouse were noted, with mice placed onto the gauzed lids of 250-mL plastic containers, with one mouse per container, with each container holding 13–15 CHIKV- or VLA1553-fed mosquitoes. There were six mice fed on six containers of mosquitoes, three mice for CHIKV-fed mosquitoes and three mice for VLA1553-fed mosquitoes. Blood samples were taken from the mice using tail bleeds to evaluate viraemia using CCID_50_ assay [43, 47]. Mice were euthanised using carbon dioxide when ethically defined endpoints were reached.

### RT-qPCR analyses of in vitro blood meals

To allow correlation of the blood meal feeding threshold titre (measured by CCID_50_ assay) to VLA1553 post-vaccination viraemia titres in humans from the phase 1 clinical trial, all in vitro blood meals from mosquito feeding were re-analysed using the same RT-qPCR as reported by Wressnigg et al. [25] with a readout in genome copy equivalents (GCE)/mL. VLA1553 RNA extraction from sheep blood was performed using the QIAamp Viral RNA Mini Kit according to the manufacturer’s instructions. For RT-qPCR, the iTaq™ Universal Probes One-Step Kit (Bio-Rad) was used. The CHIKV specific primers and the FAM-BHQ1 probe used have previously been published [52]. A qualified in vitro CHIKV RNA standard (CHIKV Δ5nsP3, VLA1553) was included for quantification. The RNA was reverse transcribed into cDNA by incubation at 50 °C for 10 min, followed by a 60-s denaturation step at 95 °C for inactivation of the reverse transcriptase and activation of the DNA polymerase. Amplification was performed in 45 cycles of denaturation at 95 °C for 10 s and annealing/extension at 61 °C for 30 s.

The LOD and limit of quantification (LOQ) were defined as 1087 GCE/mL and 3261 GCE/mL, respectively. The results were evaluated with Bio-Rad CFX Maestro software (version 4.0.2325.0418).

### Statistics

Statistical analyses were performed with GraphPad Prism (version 10.1.0). For multiple comparisons, the Kruskal–Wallis and Dunn’s multiple-comparison tests were used. For analysing differences between CHIKV and VLA1553 titres, the Mann–Whitney *U* exact test was used. Spearman’s correlations (rho and *P* values) were undertaken using IBM SPSS Statistics 23.0.

## Results

### Experimental set-up

In the first series of experiments (series 1), a comparison was undertaken between CHIKV and VLA1553 transmission to *A. albopictus* mosquitoes using in vitro blood meals containing high viral titres of ~ 7–8 log_10_CCID_50_/mL. In a second series of experiments (series 2), we sought to determine the minimum threshold blood meal VLA1553 titre required for infection, dissemination and detection of VLA1553 in mosquito saliva expectorates as a determinant of further transmission to a new mammalian host. For both series, independent experiments were performed in triplicate, which yielded a total of 173–232 fed mosquitoes per tested blood meal titre (see totals in Table 1).

**Table 1:** Mosquito membrane feeding rates showing total number of mosquitoes, number of fed mosquitoes and percentage of fed mosquitoes for CHIKV and VLA1553 blood meals

Experiment	Repeat	Titre (log_10_	Number of mosquitoes		Number of fed mosquitoes		Percentage of fed mosquitoes	
		CCID_50_/mL)	VLA1553	CHIKV	VLA1553	CHIKV	VLA1553	CHIKV
Series 1	1.1	8.38	102	144	45	42	44.1	29.2
	1.2	7.90	236	236	23	54	9.8	22.9
	1.3	7.25	383	367	120	136	31.3	37.1
					Total 188	Total 232	Mean 28.4	Mean 29.7
Series 2	2.1	5.65	150	NA	23	NA	15.3	NA
	2.2	5.75	254	NA	96	NA	37.8	NA
	2.3	5.75	233	NA	82	NA	35.2	NA
					Total 201		Mean 29.4	
	2.1	4.50	136	NA	30	NA	22.1	NA
	2.2	4.75	282	NA	76	NA	27.0	NA
	2.3	4.50	261	NA	87	NA	33.3	NA
					Total 193		Mean 27.5	
	2.1	3.875	169	NA	39	NA	23.1	NA
	2.2	3.75	251	NA	55	NA	21.9	NA
	2.3	3.75	230	NA	79	NA	34.4	NA
					Total 173		Mean 26.5	

Series 1: CHIKV versus VLA1553 blood meals, CHIKV blood meals in repeat experiments 1.1, 1.2 and 1.3 were 8, 7.38 and 7.13 CCID_50_/mL, respectively

Series 2: VLA1553 threshold blood meal titre determinations

NA: not applicable (series 2 experiments were not performed for CHIKV)

### Mosquito feeding rates

To assess whether the percentage of mosquitoes taking a blood meal was similar for blood meals containing CHIKV or a range of VLA1553 titres, the percentages of mosquitoes that took a blood meal of sheep blood spiked with either CHIKV or VLA1553 (mosquito feeding rates) were assessed. For VLA1553 versus CHIKV at ~ 7–8 log_10_CCID_50_/mL blood meal titres (series 1) and for the descending series of VLA1553 blood meal titres (series 2), the mean percentage of mosquitoes that took a blood meal was very similar (Table 1). The means of three replicates fell within a narrow range of 26.5–29.7% (Table 1) (mean ± SD: 28.3 ± 1.36%). Thus, neither the presence of CHIKV or VLA1553 nor the titre of VLA1553 in the blood meals significantly affected the mosquito feeding rate.

### Mosquito body infection rates and titres after CHIKV and VLA1553 blood meals

**Fig. 1:**
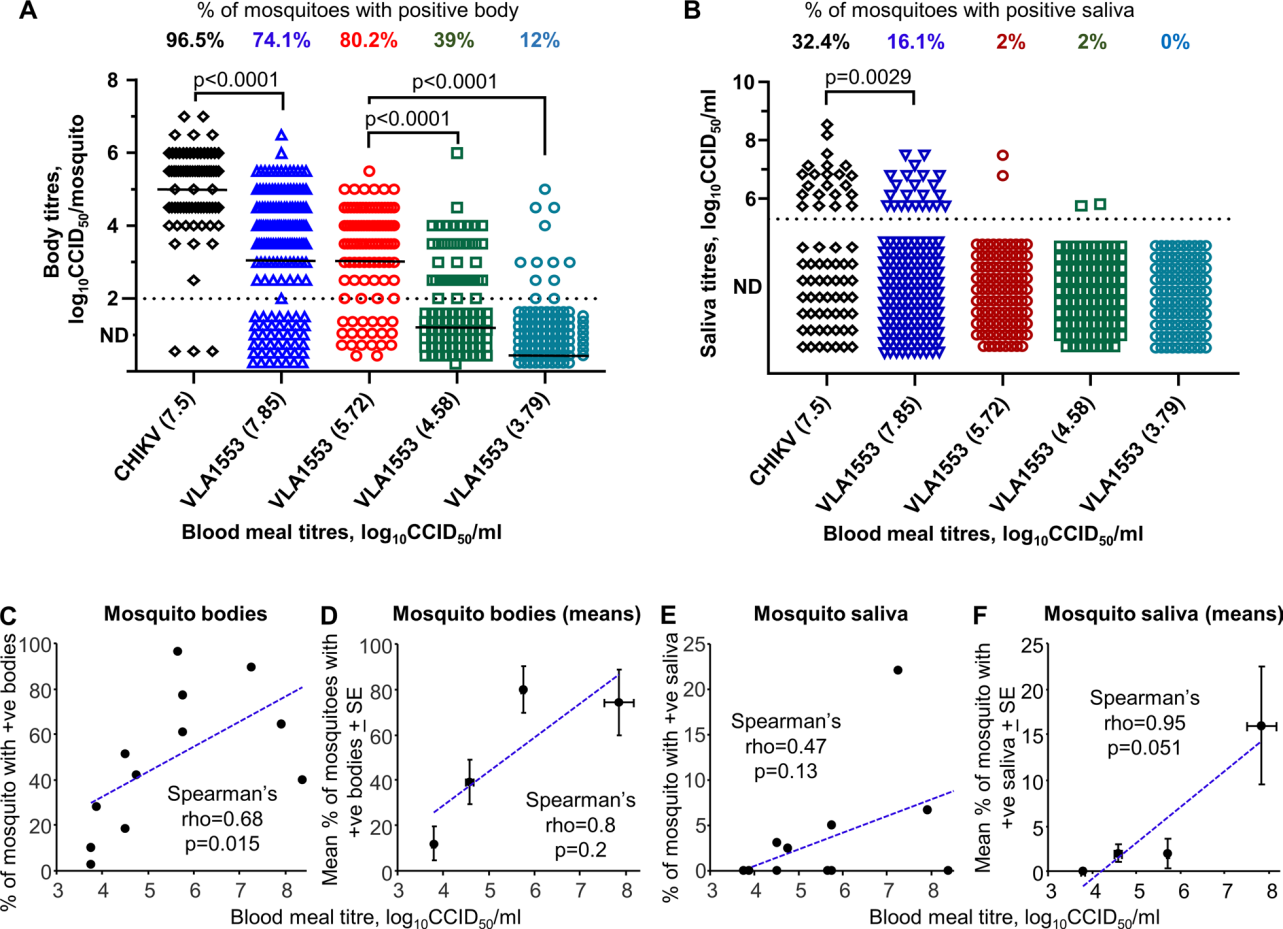
Titres in mosquito bodies and saliva after CHIKV and VLA1553 blood meals. **A** Mosquito body virus titres shown for CHIKV and VLA1553 at blood meal virus titres of ~ 7–8 log_10_CCID_50_/mL measured for series 1 experiments and body virus titres measured for series 2 experiments at descending blood meal virus titres of VLA1553. The blood meal virus titres shown in brackets on the *x* axis are the mean of three independent experiments (Table 2). The body virus titres for individual mosquitoes from the three independent experiments are shown on the *y* axis. ND, not detected; detection limit ~ 2 log_10_CCID_50_ (dashed line). Horizontal black lines represent mean titres, with ND set at zero. Statistics by Mann–Whitney *U* exact tests. **B** Mosquito saliva virus titres shown for CHIKV and VLA1553 at blood meal virus titres of ~ 7–8 log_10_CCID_50_/mL measured for series 1 experiments and saliva virus titres measured for series 2 experiments at descending blood meal titres of VLA1553. ND, not detected; saliva virus titre detection limit ~ 5.3 log_10_CCID_50_/mL saliva (dashed line). Statistics by Mann–Whitney *U* exact test. **C** Correlation between the percentage of mosquitoes with detectable VLA1553 present in the mosquito bodies versus the blood meal virus titre using the full data set in Table 2. **D** Correlation between the mean percentage of mosquitoes with detectable VLA1553 present in the mosquito bodies versus the mean blood meal titre (Table 2). **E** Correlation between the percentage of mosquitoes with detectable VLA1553 present in the mosquito saliva versus the blood meal virus titre using the full data set in Table 2. **F** Correlation between the mean percentage of mosquitoes with detectable VLA1553 present in the mosquito saliva versus the mean blood meal virus titre (Table 2)

A mean of 96.5% (84/87) of mosquitoes that fed on blood meals containing ~ 7–8 log_10_CCID_50_/mL of CHIKV developed virus-positive bodies over three independent experiments. For VLA1553 these numbers were 74.1% (120/162) (Table 2, series 1, Fig. 1A).

**Table 2:** Post-blood meal body and saliva positivity rates for wild-type CHIKV- and VLA1553-fed mosquitoes

Group	Experiment	Repeat	Blood meal virus titre (log_10_CCID_50_/mL)	Body			Saliva		
				Mosquitoes tested for body titres (*n*)	Virus positive (*n*)	Body positive (%)	Fed mosquitoes tested for saliva titres (*n*)	Saliva positive (*n*)	Saliva positive (%)
VLA1553	Series 1	1.1	8.38	42	17	40.5	28	0	0.0
		1.2	7.92	20	13	65.0	15	1	6.7
		1.3	7.25	100	90	90.0	100	22	22.0
			Mean 7.85	Sum 162	Sum 120	Mean 74.1%*	Sum 143	Sum 23	Mean 16.1%*
	Series 2	2.1	5.65	35	34	97.1	34	0	0.0
		2.2	5.75	40	31	77.5	40	2	5.0
		2.3	5.75	26	16	61.5	26	0	0.0
			Mean 5.72	Sum 101	Sum 81	Mean 80.2%*	Sum 100	Sum 2	Mean 2%*
		2.1	4.50	28	16	51.7	28	0	0
		2.2	4.75	40	17	42.5	40	1	2.5
		2.3	4.50	32	6	18.8	32	1	3.1
			Mean 4.58	Sum 100	Sum 39	Mean 39%*	Sum 100	Sum 2	Mean 2%*
		2.1	3.88	21	6	28.6	21	0	0.0
		2.2	3.75	48	5	10.4	48	0	0.0
		2.3	3.75	31	1	3.2	31	0	0.0
			Mean 3.79	Sum 100	Sum 12	Mean 12%*	Sum 100	Sum 0	Mean 0%*
CHIKV	Series 1	1.1	8	41	40	97.6	29	12	41.4
		1.2	7.38	26	25	96.2	19	4	21.1
		1.3	7.13	20	19	95.0	20	6	30.0
			Mean 7.50	Sum 87	Sum 84	Mean 96.5%*	Sum 68	Sum 22	Mean 32.4%*

Data are derived from experiments comparing CHIKV and VLA1553 at ~ 7–8 log_10_CCID_50_/mL blood meal virus titres (series 1 with repeat experiments 1.1, 1.2 and 1.3) and from experiments testing VLA1553 at descending titres (series 2 with repeat experiments 2.1, 2.2 and 2.3). *All mean percentages (body and saliva) calculated from summed numbers, not mean of percentages

The body virus titres after CHIKV and VLA1553 blood meals containing ~ 7–8 log_10_CCID_50_/mL of virus were significantly different, with VLA1553-fed mosquitoes showing a ~ 1.9 log lower mean body virus titre (Fig. 1A, *P* < 0.0001 by Mann–Whitney *U* exact test). However, owing to the high number of not detected (ND), set to zero for statistical treatments resulting in ties, use of a rank test might be viewed as providing unreliable results, although a non-parametric test is clearly required given the data distributions. Nevertheless, the VLA1553-fed group also contained a significantly higher proportion of ND when compared with the CHIKV-fed group (chi-squared tests), supporting the contention of a significant difference between these groups.

For VLA1553-fed mosquitoes, as the blood meal virus titres were reduced, the percent of mosquitoes with positive bodies decreased, and mean body virus titres also significantly declined (Table 2, Fig. 1A, *P* < 0.0001). The Mann–Whitney *U* exact test results are supported by chi-squared tests, which show a significant increase in the number of ND as the VLA1553-titres in the blood meals were reduced (*P* < 0.0001 for both comparisons).

### Saliva infection rates and titres after CHIKV versus VLA1553 blood meals

For the three series 1 experiments for mosquitoes that fed on blood meals with a mean CHIKV titre of 7.5 log_10_ CCID_50_/mL, 32.4% (22/68) of mosquitoes had virus-positive saliva (Table 2, Fig. 1B). In contrast, for VLA1553, at a similar mean blood meal virus titre of 7.85 log_10_ CCID_50_/mL, 16.1% (23/143) of mosquitoes had virus-positive saliva (Table 2, Fig. 1B). Across the three repeat experiments, the percentages of mosquitoes with virus-positive saliva appeared to be generally lower in VLA1553-fed versus CHIKV-fed mosquitoes (Table 2); however, this did not reach significance (0%, 6.7% and 22% versus 41.4%, 21.1% and 30%; *P* = 0.07, *t* test for these *n* = 3 versus 3 percentages).

The virus titres in saliva were significantly lower for VLA1553-fed versus CHIKV-fed mosquitoes (Fig. 1B, *P* = 0.0029, Mann–Whitney *U* exact test). Mean titres are not provided given the large number of ND and the high limit of detection (Fig. 1B), with a non-parametric rank-based statistical method applied (Mann–Whitney *U* exact test). The contention of a significant difference is supported by a significantly higher proportion (frequency of categories) of ND in the VLA1553-fed group versus the CHIKV-fed group (chi-squared *P* = 0.0058).

### VLA1553 transmission to mosquitoes at descending blood meal virus titres

As VLA1553 was able to be transmitted to mosquitoes via an artificial blood meal, we sought to determine whether a threshold blood meal virus titre could be established for mosquito infection. A significant correlation between blood meal virus titres and mosquito body positivity rates was observed for the descending titres of the VLA1553 blood meal (Table 2, Fig. 1C, rho = 0.68, *P* = 0.015). When mean blood meal virus titres and mean percent of positive mosquito bodies were compared, rho increased, but significance was lost (Table 2, Fig. 1D, rho = 0.8, *P* = 0.2).

A correlation between blood meal virus titres and mosquito saliva positivity rates was observed for the descending titres of VLA1553 blood meal titres, but this did not reach significance (Table 2, Fig. 1E, rho = 0.47, *P* = 0.13). However, when mean blood meal virus titres and mean mosquito saliva positivity rates were compared, rho increased and the correlation approached significance (Table 2, Fig. 1F, rho = 0.95, *P* = 0.051). As no VLA1553 dissemination into saliva was observed at a blood meal virus titre of 3.875 log_10_CCID_50_/mL or below (Fig. 1B, E), this titre might thus be viewed as an approximate blood meal titre threshold for dissemination into saliva.

### Mosquito transmission of CHIKV and VLA1553 to ***Irf3/7***^−/−^ mice

Next, we wanted to determine whether mosquitoes that had fed on high -titre (~ 7–8 log_10_CCID_50_/mL) CHIKV or VLA1553 blood meals were able to transmit the wild-type virus or the vaccine to *Irf3/7*^−/−^ mice. Using mosquitoes from series 1.1 experiments, *Irf3/7*^−/−^ mice were anaesthetised and presented to mosquitoes that had taken artificial blood meals of CHIKV or VLA1553 8 days previously (*n* = 3 mice per group). The number of engorged mosquitoes (i.e. mosquitoes that had taken a blood meal from the mice) was noted for each mouse. A total of 11 of 41 CHIKV-fed and 13 of 42 VLA1553-fed mosquitoes took a blood meal from the *Irf3/7*^−/−^ mice (Table 3).

**Table 3:** Transmission of CHIKV or VLA1553 from *A. albopictus* mosquitoes to *Irf3/7*^−/−^ mice

Group	Experiment	No. of fed	Body positive	Saliva positive	No. of mosquitoes fed on mice		No. of mice with viraemia*
		mosquitoes	(%)	(%)	Total	Per mouse^#^	
VLA1553	1.1	42	40.5	0	13	5 + 3 + 5	0/3
	1.2	20	65.0	6.7	4	1 + 1 + 2	0/3
CHIKV	1.1	41	97.6	41.4	11	4 + 4 + 3	3/3
	1.2	26	96.2	21.0	6	2 + 2 + 2	0/3

^*^Read out for infection was viraemia measurements from day 0 to day 16 by CCID_50_ assays [43] (see Fig. 2). ^#^One mouse per container of CHIKV or VLA1553-fed mosquitoes (13–15 per container), *n* = 3 mice per group

Experiment: series 1 with CHIKV versus VLA1553 using mosquitoes from repeat experiments 1.1. and 1.2

**Fig. 2:**
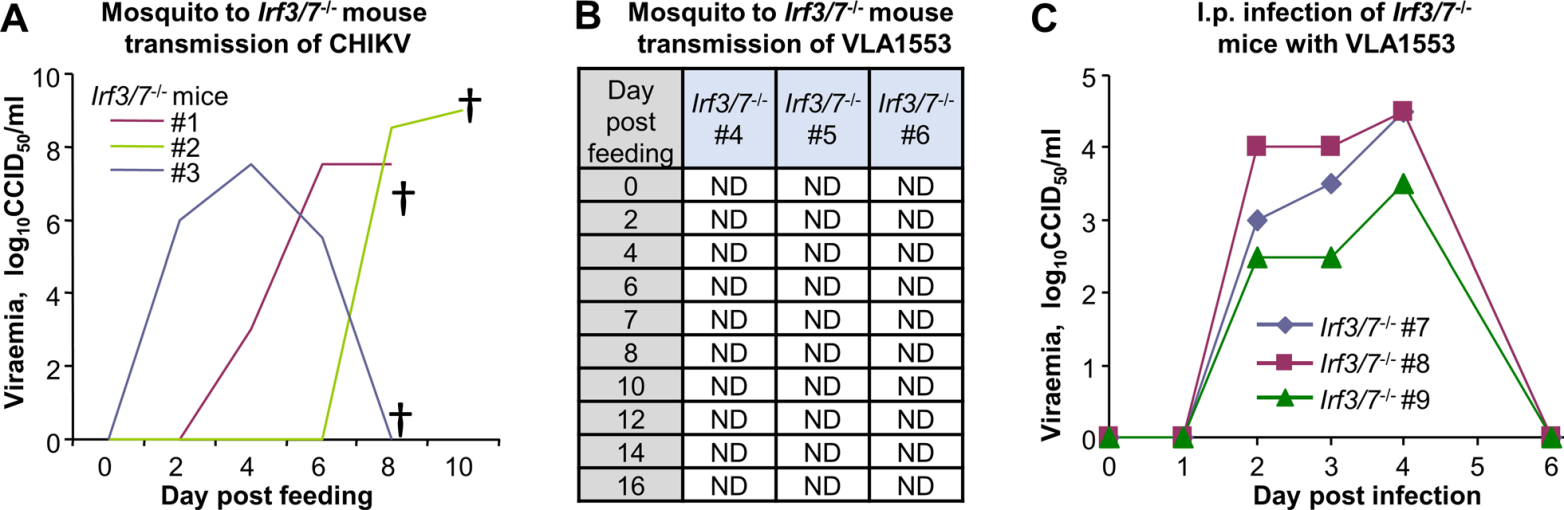
Infection of *Irf3/7*^−/−^ mice. **A** CHIKV-fed mosquitoes were allowed to feed on three *Irf3/7*^−/−^ mice, and transmission of virus from mosquitoes to mice was assessed by viraemia determinations using CCID_50_ assays of serum samples. The viraemia for each individual mouse (#1, #2 and #3) is plotted. †Mice reached ethically defined endpoints for euthanasia. See Table 3 for details. **B** VLA1553-fed mosquitoes were allowed to feed on *Irf3/7*^−/−^ mice, and transmission was assessed by viraemia determinations using serum from *Irf3/7*^−/−^ mice and CCID_50_ assays. ND, not detected; limit of detection was ~ 2 log_10_CCID_50_/ml. See Table 3 for details. **C** To confirm that *Irf3/7*^−/−^ mice can be infected with VLA1553, three mice were inoculated via the intraperitoneal route with 4 log_10_CCID_50_ of VLA1553 and individual viraemia determined by CCID_50_ assays as for **A**

All three mice exposed to CHIKV-fed mosquitoes developed CHIKV viraemia and reached ethically defined endpoints for euthanasia (Fig. 2A; Table 3). The different times for the onset of viraemia for the three mice (Fig. 2A) may reflect different virus inoculum doses, with inoculation of low viral titres perhaps resulting in a longer delay before detectable viraemia is established. In contrast to the results in Fig. 2A, none of the mice exposed to VLA1553-fed mosquitoes developed detectable viraemia (Fig. 2B; Table 3). We confirmed that *Irf3/7*^−/−^ mice, infected with VLA1553 via the intraperitoneal route, developed readily detectable viraemia (Fig. 2C), with peak viraemia ~ 5.5–6.5 logs lower than those seen after infection of *Irf3/7*^−/−^ mice with 4 log_10_CCID_50_ of CHIKV [43]. VLA1553-infected mice showed no or mild symptoms and did not reach ethically defined endpoints requiring euthanasia. In a repeat experiment using series 1.2 mosquitoes, none of the mice developed detectable viraemia (Table 3). These experiments provide some in vivo support for the contention that the low percentage of VLA1553-fed mosquitoes with positive saliva by CCID_50_ assays (0% and 6.7%, limit of detection ~ 5.3 log_10_ CCID50/mL; Table 2) translates to poor transmission to a highly sensitive mouse strain.

### Comparison of threshold titre for VLA1553 transmission to viraemia in vaccinees from the phase 1 clinical trial

To assess the risk of VLA1553 being transmitted from viraemic, vaccinated individuals to mosquitoes, we determined the mean conversion for log_10_CCID_50_/mL (used to determine blood meal titres herein) to GCE/mL (used to determine viraemia in vaccine recipients in clinical trials). As shown in Table 4, the correlation between blood meal virus titres and RT-qPCR titres resulted in a mean ± SD conversion factor of 250 ± 35.4, with no indication that the blood meal virus dose significantly affected the conversion factor; the relationship was essentially linear across 3 logs. Thus, the threshold titre of 3.875 log_10_ CCID_50_/mL (described above) for transmission corresponds to ~ 6.27 log_10_GCE/mL.

**Table 4:** Correlation between blood feeding virus titres (CCID_50_/mL) and RT-qPCR titres (GCE/mL) derived from series 2 experiments

	Blood feeding virus titre 5.72 log_10_ CCID_50_/mL			Blood feeding virus titre 4.58 log_10_ CCID_50_/mL			Blood feeding virus titre 3.79 log_10_ CCID_50_/mL		
	Exp. 2.1*	Exp. 2.2*	Exp. 2.3^†^	Exp. 2.1*	Exp. 2.2*	Exp. 2.3^†^	Exp. 2.1*	Exp. 2.2*	Exp. 2.3^†^
Pre-feeding titre (log_10_CCID_50_/mL)	5.75	5.75	5.75	4.5	4.75	4.75	4.25	4.00	4.00
Post-feeding titre (log_10_CCID_50_/mL)	5.50	5.75	5.75	4.5	4.75	4.25	3.50	3.50	3.50
RT-qPCR run 1 (GCE/mL)^∞^	1.30E + 08	1.90E + 08	1.77E + 08	1.46E + 07	1.76E + 07	1.89E + 07	9.15E + 05	2.00E + 06	1.44E + 06
RT-qPCR run 2 (GCE/mL)^∞^	3.24E + 08	3.12E + 08	3.48E + 08	3.68E + 07	3.49E + 07	3.57E + 07	2.44E + 06	2.75E + 06	2.91E + 06
RT-qPCR run 3 (GCE/mL)^∞^	2.48E + 08	2.72E + 08	3.41E + 08	2.50E + 07	2.99E + 07	3.22E + 07	1.89E + 06	2.84E + 06	2.59E + 06
Average GCE/mL	2.34E + 08	2.58E + 08	2.89E + 08	2.55E + 07	2.75E + 07	2.89E + 07	1.75E + 06	2.53E + 06	2.31E + 06
Factor CCID_50_/mL versus GCE/mL	233.95	257.96	288.69	254.78	274.9	289.12	174.66	252.71	231.19
Average recovery control*	2.31E + 08			1.87E + 07			2.09E + 06		

Sheep blood freshly spiked with VLA1553 at respective CCID_50_/mL

^†^0.5 h feeding; *1 h feeding; ^∞^LOD_95%_ ≥ 10 GCE/reaction (2.5 × 10^3^ GCE/mL); CCID_50_, 50% cell culture infectivity dose; Exp, experiment; GCE, genome copy equivalents

## Discussion

VLA1553 is a live-attenuated vaccine that has a reduced replication capability due to a 61-amino-acid deletion in the nsP3 protein of the replicase complex [24]. We aimed to assess whether the attenuated profile of the arbovirus would affect the potential for transmission from blood meals to mosquitoes, dissemination into the saliva and transmission from mosquitoes to mice. We also compared this data with previous clinical trial data to assess the potential for mosquito-based transmission of VLA1553 from vaccine recipients to other humans.

First, when looking at the transmission of CHIKV from artificial blood meals to *A. albopictus* mosquitoes, differences in the behaviour of VLA1553 compared with CHIKV were observed. At high blood meal virus titres (~ 7–8 log_10_CCID_50_/mL), the infectious titre in mosquito bodies was significantly lower for VLA1553, with a mean difference of ~ 1.9 log (Fig. 1A). A lower percentage of mosquitoes with positive saliva was also observed for VLA1553-fed mosquitoes (16.1%) compared with CHIKV-fed mosquitoes (32.4%), with virus titres significantly lower (Fig. 1B, *P* = 0.0029).

Second, as mosquito transmission of CHIKV requires dissemination to, replication within and release from the salivary glands, the presence of CHIKV and VLA1553 in mosquito saliva was assessed. A relationship between blood meal titres and the potential for onwards transmission by mosquitoes has been explored in a number of systems [33, 36–39]. Consistent with these studies, we also show herein a relationship between blood meal titres of VLA1553 and the number of mosquitoes with positive saliva. No VLA1553-positive saliva samples were detected at a blood meal VLA1553 titre of 3.875 log_10_CCID_50_/mL (Fig. 1B), which might therefore be viewed as a threshold below which dissemination to saliva is improbable (Fig. 1E, F).

Third, for the assessment of transmission of CHIKV from mosquitoes to mice, infection of *Irf3/7*^−/−^ mice by mosquitoes fed on VLA1553 blood meals was not observed, whereas for CHIKV-fed mosquitoes, transmission to mice could be clearly demonstrated. In an earlier study that investigated CHIKV transmission from *A. albopictus* mosquitoes to laboratory mice and included lower CHIKV blood meal titres [33], CHIKV titres in blood meals ≥ 7 log_10_CCID_50_/mL were required before salivary glands showed significant levels of immunofluorescent staining with an anti-CHIKV antibody. In the earlier work, mosquitoes fed on blood meals of 7.5 (but not 5.9) log_10_CCID_50_/mL were able efficiently to transmit virus to mice [33].

Lastly, we assessed the potential of transmission of VLA1553 from viraemic vaccine recipients via mosquitoes to other humans using viraemia data from clinical trials. In a phase 1 clinical trial [25], in the medium-dose group (3.2 × 10^4^ TCID_50_), viraemia was short-lived and peaked at day 3, with mean viraemia of 8.9 × 10^4^ GCE/mL (4.95 log_10_GCE/mL), which is approximately 20-fold below the threshold titre for transmission determined at 6.27 log_10_GCE/mL, assuming that sheep versus human blood does not significantly influence the conversion factor of 250 ± 35.4. The highest viraemia was 1.88 × 10^6^ GCE/mL in the phase 1 clinical trial at the medium-dose group, which would correspond to 3.87 log_10_CCID_50_/mL using the conversion factor, and is equivalent to the determined threshold titre for transmission of VLA1553 from a blood meal to the mosquito (i.e. 3.875 log_10_CCID_50_/mL). Thus, mosquitoes could potentially transmit VLA1553. However, the actual dose of licensed VLA1553 (not less than 3 log_10_TCID_50_, per the IXCHIQ package insert [27]) is lower than the dose used in the phase 1 clinical trial. Furthermore, the mosquito body titres after VLA1553 blood meals were ~ 1.9 log lower than for CHIKV. Such differences would arguably reduce dissemination to salivary glands [32]. Our data support this contention, with 16.1% versus 32.4% of mosquitoes showing positive saliva after VLA1553 and CHIKV blood meals, respectively. The short-lived nature of the VLA1553 viraemia would also restrict the number of days on which the threshold titre is reached in the blood. In addition, host-seeking behaviour of mosquitoes involves sensing of infrared radiation emanating from body heat [53], and VLA1553 vaccinations are generally not associated with the elevated body temperature (fever) [26] that is usually seen after CHIKV infection [2]. Finally, acetophenone from flavivirus-infected animals was recently reported as a potent chemoattractant for blood-seeking mosquitoes. Flavivirus infection suppressed expression of skin resistin-like molecule alpha (RELMα), leading to proliferation of acetophenone-producing skin bacteria [54]. It would be interesting to determine whether CHIKV infection had the same activity, and whether such acetophenone production was lower after infection with VLA1553. Thus, although the peak VLA1553 viraemia in vaccine recipients reaches the threshold, other factors may mitigate against efficient transmission.

Limitations of our study include the lack of CHIKV transmission data for lower blood meal virus titres, although previously published data with lower titres [33] complement our results for mosquito body and saliva positivity. The feeding behaviour and transmission characteristics of mosquitoes in the wild and in laboratory settings may differ [55–61], thereby limiting the validity of the extrapolation of our data to real-world situations. Nevertheless, feeding on humans in the laboratory has been shown to be reasonably well mimicked by membrane feeding in the laboratory [62].

## Conclusions

Our study showed differences in terms of CHIKV transmission in mosquitoes fed VLA1553 versus CHIKV, with less efficient infection of mosquitoes for VLA1553 than for CHIKV, no subsequent transmission to nor infection of mice by mosquitoes infected by VLA1553 and no virus-positive saliva below a VLA1553 blood meal threshold titre of 3.875 log_10_CCID_50_/mL. The latter titre also corresponds to the maximum titre associated with viraemia in vaccine recipients. Overall, mosquito-borne transmission of VLA1553 from vaccinated individuals is considered to be a low-probability event.

## Data Availability

Data are provided within this manuscript. Materials may be requested from the corresponding author.
